# Hydrodynamic cavitation assisted recovery of intracellular polyhydroxyalkanoates

**DOI:** 10.1007/s00449-025-03197-3

**Published:** 2025-07-06

**Authors:** Tülin Yilmaz Nayir, Yusuf Küçükağa, Serdar Kara

**Affiliations:** Department of Environmental Engineering, Gebze Technical University, 41400 Gebze, Kocaeli Turkey

**Keywords:** Bioplastic, PHA, PHB, Hydrodynamic cavitation, Biopolymer extraction, Mixed microbial culture

## Abstract

In this study, the hydrodynamic cavitation (HC) process was adopted for the recovery of intracellular biopolymer, namely polyhydroxyalkanoates (PHAs), from mixed microbial culture (MMC). To investigate the potential and performance of HC process, two cavitation devices (orifice-1 and orifice-17) were employed. The impact of biomass concentration, orifice type and pressure differential on recovery yield was assessed. The HC-assisted PHA recovery protocol introduced a novel technique that uses HC for cell disruption and a solvent for biopolymer separation. The results demonstrate the feasibility of obtaining biopolymer within a short operation time (5 min), achieving 72% process efficiency using the HC-assisted recovery procedure. The biopolymer recovered via HC at optimal conditions exhibited a purity of 71.4%, indicating effective polyhydroxybutyrate (PHB) isolation. Its molecular weight of 0.15 × 10⁶ g/mol aligns with typical PHB ranges, suggesting its suitability for various applications. Fourier-transform infrared spectroscopy (FTIR) analysis confirmed compatibility with commercial PHB. Thermal degradation profiles showed slightly lower stability compared to commercial PHB, with a 10% mass loss at 243.21 °C and a maximum degradation temperature of 262.12 °C. Despite these minor differences, HC presents a promising, greener method for PHA recovery, offering potential applications in sustainable industries.

## Introduction

PHAs are a class of biodegradable polymers naturally produced by diverse microorganisms as intracellular carbon and energy reserves. With growing environmental concerns over plastic waste, PHAs have gained global attention as promising alternatives for conventional, petroleum-based plastics. PHAs find applications in disposable tableware and packaging materials, shopping bags, and agricultural mulch films [1]. However, despite projected production growth to 570 kilotons per year by 2027, commercial PHA adoption remains limited, primarily due to high production costs [2]. The commercial production of PHAs typically incurs costs ranging from approximately $2.24 to $5.09 per kilogram, notably 5–10 times higher than the traditional plastics, typically priced at less than $1.02 per kilogram [3]. A significant portion of this cost arises from downstream processing, particularly the recovery of PHAs from microbial biomass. This step not only impacts overall production economics but also determines the purity and usability of the final biopolymer. Thus, developing efficient, scalable, and environmentally friendly PHA recovery methods are essential for improving the viability of this sustainable material.

Traditional PHA recovery techniques, including solvent-based extraction, mechanical disruption, and enzymatic digestion, have been widely studied. Solvent-based methods, though efficient, rely on large volumes of often-toxic chemicals, limiting scalability [4, 5]. Emerging alternatives such as supercritical fluid extraction show promise with high recovery efficiency and environmental benefits but involve complex setups and high operational pressures [6, 7].

Mechanical disruption techniques, such as ultrasound, bead milling, and high-pressure homogenization, expose microbial cells to significant physical stress, facilitating PHA release [8].

Several studies have evaluated mechanical disruption for PHA recovery. Ultrasound irradiation has been employed to enhance PHA extraction [9]. Tamer et al. (1998) compared mechanical (bead milling and high-pressure homogenization) and chemical disruption (sodium dodecyl sulfate and sodium hypochlorite) in *Alcaligenes latus*, showing that bead milling achieved rapid cell disruption levels comparable to chemical treatment, which released over 90% of cellular protein [10]. High-pressure homogenization, when used as a pretreatment for solvent extraction, achieved a PHA recovery yield of 51% [11]. Other studies have reported a maximum yield and purity of 95% and 80%, respectively, at an operating pressure of 400 kg/cm^2^ [12]. In high-density *Cupriavidus necator* cultures (~ 200 g/L PHA-rich biomass), successful cell disruption was demonstrated at 800 kg/cm^2^ [13].

However, mechanical techniques often introduce undesirable impurities such as fine cell fragments, proteins, enzymes, and nucleic acids, requiring additional purification steps. The search for a more selective and scalable approach has led to the exploration of cavitation-assisted cell disruption as a promising alternative [14, 15]. Cavitation is defined as the formation of small vapor bubbles in a homogeneous medium triggered by rapid pressure drop [16]. It is broadly classified in two types: acoustic and hydrodynamic. In acoustic cavitation, bubble formation occurs due to the high frequency of acoustic waves. In contrast, HC is induced by pressure fluctuations in a flowing fluid system, typically caused by a constriction, such as a venturi tube or an orifice plate [17]. The rapid collapse of cavitation bubbles generates intense localized energy, releasing free radicals that facilitate cell disruption and component extraction [18].

In this context, HC emerges as a novel and transformative technology for PHA recovery. The versatility of HC has been demonstrated across a wide range of applications, from food processing and waste remediation [19, 20] to the extraction of bioactive compounds, essential oils, and pigments [21]. It has been effectively employed in biomass processing, such as oil extraction from wet microalgae [22, 23], pectin recovery [24], and microbial cell disruption for intracellular product recovery [25]. However, to the best of authors knowledge, its potential for the recovery of intracellular PHA granules has not been studied until now.

In this study, the HC process was employed for the recovery of biopolymers from MMC-based biomass, demonstrating efficient performance within a short operation time. This novel technique, applied for the first time to the recovery of intracellular PHA granules, was evaluated for its effectiveness on different biomass concentrations, orifice types, and pressure levels in relation to product yield. The chemical and thermal properties of the recovered biopolymer were also examined, including its molecular weight, purity, FTIR profile, and thermogravimetric analysis (TGA) results.

## Material and methods

### Microbial culture and PHA accumulation

PHA accumulation experiments were conducted in a 3L sequencing batch reactor (SBR) over a 3-month period, employing MMC subjected to a feast-famine regime to acclimatize PHA-accumulating microbial consortia, as described in detail elsewhere [26]. Anaerobically pretreated yeast industry wastewater (YWW) was used as the substrate in the biological SBR. The resulting PHA-enriched slurry was centrifuged at 9000 rpm for 5 min, followed by freeze-drying. The dried biomass -predominantly composed of PHB at a content of 35%-, was used in all subsequent experiments.

### Solvent extraction

The intracellular biopolymer was extracted using chloroform as a conventional method, in addition to HC-assisted recovery, to enable a comparative evaluation of both extraction approaches in terms of recovery yield and polymer characterization. 100 mg of freeze-dried MMC biomass was extracted with 4 mL of chloroform for 1 h in a closed tube. Then the solution was centrifuged at 3000 rpm for 1 min and filtered with polypropylene membrane filters of 0.45 μm porosity. The polymer was recovered by solvent evaporation and then dried under a fume hood for 24 h.

### Hydrodynamic cavitation process

**Fig. 1:**
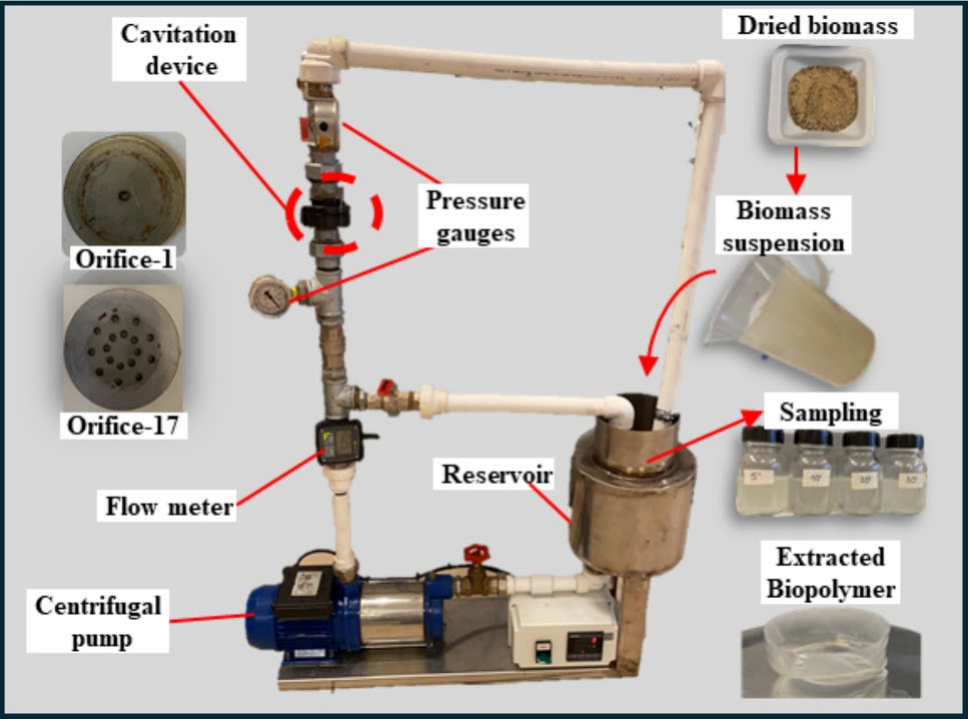
Schematic of the experimental setup used for HC-assisted PHA recovery

The experimental set-up used in this study is shown in Fig. 1. Such process was validated by repeated cavitation tests of PHA-containing microbial suspensions. These preliminary tests confirmed the replicability of the procedure with an acceptable precision of recovery efficiencies (± 10%). The HC reactor comprises a centrifugal pump (SUMAK, SM15) for fluid flow and pressure, a 3 L stainless-steel reservoir tank with a cooling chamber, pressure gauges, a flow meter (FMT 3, Germany), and the cavitation device. The design of the cavitation device was guided by insights from prior literature on hydrodynamic cavitation reactor geometry. Previous studies have highlighted that single-hole orifices can produce more intense cavitation effects due the higher velocity and greater pressure drop per unit area, which are critical for generating effective cavitation [17]. Based on this, two cavitation plates were designed with a radial hole pattern: one featuring a single 1 mm diameter hole (orifice-1), and the other containing seventeen 1 mm diameter holes (orifice-17)—the maximum number feasible on the plate. This configuration enabled a systematic investigation of how variations in free flow area influence cavitation performance, specifically in terms of biopolymer recovery efficiency.

Biomass suspensions with varying solid loads (g/L) were placed into the reservoir tank. The pressure was regulated with a valve, and the biomass suspension was directed through the orifice. Optimum recovery efficiency was investigated under different operating conditions, including cavitation plate type (orifice-1 and orifice-17), solid loading (0.5–2 g/L), and pressure input (2–6 bar). During the operation, 20 mL samples were collected from the reservoir at 5-, 10-, 20-, and 30-min of operation time. Biomass samples disrupted by HC were transferred to glass tubes, where 5 mL of chloroform (Supelco^®^, analytical grade) was added to facilitate PHA solubilization. After vigorous shaking, the tubes were centrifuged at 9000 rpm for 5 min to allow phase separation. The polymer was subsequently recovered by evaporating the chloroform and drying the residue under ambient conditions in a fume hood.

### Chemical and thermal analysis

PHA content in the biomass was quantified with a gas chromatography (GC-FID Agilent) procedure [26]. GC is configured with a Flame Ionization Detector (FID) set to 275 °C and a Carbowax column measuring 30 m in length and 0.53 mm in diameter. Helium is employed as the carrier gas, flowing consistently at a rate of 3 mL/min. The temperature protocol is as follows: initially held at 80 °C for 1 min, followed by a ramp of 20 °C/min up to 160 °C; then, after 1 min from the start of the program, there is an oven temperature increase of 10 °C/min up to 240 °C, where it is maintained for 2 min. Operating in 8:1 split mode, the inlet temperature is set to 230 °C. This method involves digesting 10 mg of biomass with 2 mL chloroform and 2 mL of digestion solution in a high-pressure vial at 100 °C for 3.5 h in a thermo-reactor. For the analysis of polymer purity, 5 mg of the recovered biopolymer was subjected to the same GC-FID method.

The molecular structure and functional groups of the recovered biopolymer were characterized using Fourier Transform Infrared Spectroscopy (PerkinElmer Spectrum 100 FT-IR spectrophotometer). The FTIR analysis covered the wave number ranges between 400 and 4000 cm^−1^.

The thermal stability of the recovered biopolymer was assessed using the Mettler Toledo TGA/SDTA 851. The analysis was conducted at a heating rate of 10 °C/min under a nitrogen flow of 50 mL/min.

The viscosity of each sample was assessed using the AND SV-10A Vibro Viscometer equipment to determine the viscosimetric molecular weight.

### Calculations and formulas

The PHB content of the biomass was calculated according to Eq. (1): where *m*_PHB_ is the mass of PHB as quantified by GC-FID and ${m}_{\text{biomass}}$ is the  amount of digested biomass (~ 10 mg) as mg.

1 $$\text{PHB content } \left(\%\right)=\frac{{m}_{\text{PHB}}}{{m}_{\text{biomass}}}\times 100$$

Purity of recovered biopolymer as PHB was calculated according to Eq. (2): where the ${m}_{\text{PHB}}$ is the mass of PHB as quantified by GC-FID, the ${m}_{\text{biopolymer}}$ is the total mass of polymer sample used for the GC analysis.

2 $$\text{Biopolymer purity } \left(\%\right)=\frac{{m}_{\text{PHB}}}{{m}_{\text{biopolymer}}}\times 100$$

The HC-assisted recovery efficiency (*Y*) was calculated by a gravimetric method where the biopolymer obtained after each experiment was dried under fume hood for 24 h and weighed. The *Y* (%) was expressed in Eq. (3):

3 $$Y = \frac{{m_{{{\text{biopolymer}} }} \;\left( {\text{g}} \right)}}{{{\text{solid load}}\; \left( {\frac{{\text{g}}}{{\text{L}}}} \right) \times {\text{sample volume}}\;\left( {\text{L}} \right) \times {\text{PHB}}_{{{\text{biomass}}}} }}$$

Viscosimetric molecular weight was calculated according to Mark Houwink’s equation (Eq. 4): $\eta$ is the intrinsic viscosity value (dL/g), ${M}_{\text{w}}$ is molecular weight (g/mol), Mark Houwink’s parameters *K* and $\alpha$ for PHA-CHCl_3_ system are $1.18 \times 10^{ - 4}$ dL/g and 0.78 respectively [27].

4 $$\eta =K{M}_{w}^{\alpha }$$

The intrinsic viscosity ($\eta )$ values was calculated according to Solomon–Ciuta [28] in Eq. (5): where the *c* is the concentration of polymer solution (g/100 mL), $\eta$_sp_ and $\eta$_rel_ are specific and relative viscosity values, respectively. The dried polymer sample was dissolved in CHCl_3_ as concentration solution of %0.1 wt (%) under stirring for 3 h [28].

5 $$\eta =\frac{\sqrt{2}}{c}\sqrt{{\eta }_{\text{sp}}-ln{\eta }_{\text{rel}}}$$

Cavitation number (CN) was calculated according to Eq. (6); where *P*_v_ is the vapor pressure of liquid, *P*_d_ is the downstream pressure, $\rho$ is the density of the fluid, and *V* is the flow velocity [29]. Upstream pressure was 2–4–6 bar according to operation parameters and downstream pressure was 1 bar because of the open reactor system.

6 $$\text{CN}=\frac{({p}_{\text{d}}-{p}_{\text{v}})}{\frac{\rho \cdot {V}^{2}}{2}}$$

### Statistical analysis

ANOVA was performed using Minitab 22 to evaluate the influence of orifice type (*X*1, categorical), pressure (*X*2, continuous), and time (*X*3, continuous) on the biopolymer recovery yield (*Y*).

## Results and discussion

### Effect of biomass load on biopolymer recovery efficiency

**Fig. 2:**
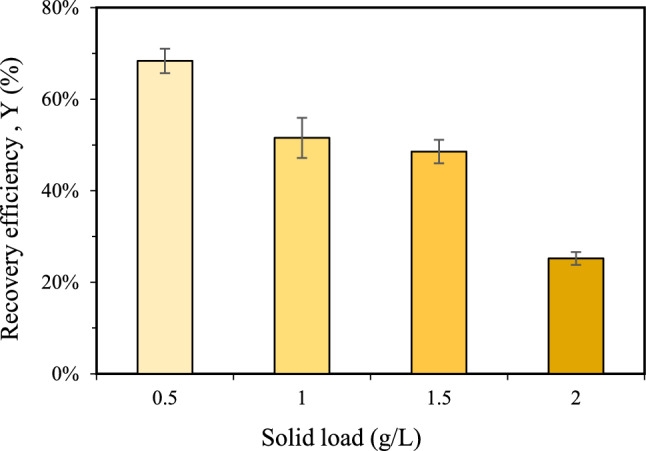
Effect of solid load on HC-assisted PHA recovery efficiency

The impact of varying biomass loadings (0.5, 1.0, 1.5 and 2.0 g/L) on the PHA recovery efficiency was investigated in the HC process employing orifice-17, under constant conditions of 4 bar pressure and 5 min operation time. As shown in Fig. 2, recovery efficiency decreased progressively with increasing biomass concentration. At 0.5 g/L biomass load, recovery efficiency of 68% was achieved. However, a substantial decline to 25% was observed at 2.0 g/L, indicating an inverse relationship between biomass concentration and recovery efficiency. This decrease can be attributed to the non-uniform impact of cavitation bubbles on microbial cells, leading to inefficient cell disruption at higher biomass concentrations. Increased solid content alters sludge flow dynamics, impeding cavitation formation and thereby reducing the efficiency of cell disruption and biopolymer release [23, 30]. In agreement with previous studies, low solid loads have been identified as optimal for HC-assisted PHA recovery [23, 31]. Similar trends have been observed in other extraction studies where the increasing initial cell concentration reduced the amount of protein released due to decreased availability to the number of cavitation bubbles available to each cell [32, 33]. Although the experimental results indicate that HC-assisted process shows higher recovery performance at lower biomass concentrations, this characteristic may increase operational costs in industrial applications due to the need for higher water volumes and greater energy input for subsequent processing steps. Therefore, operating at higher biomass concentrations is essential to enhance the overall cost-efficiency of the recovery process. To address this limitation, HC system designs—such as vortex-based cavitation device—should be considered, as recent studies shown their potential to maintain cavitation efficiency at higher biomass loadings [34].

### Effect of pressure input on HC-assisted recovery efficiency

**Fig. 3:**
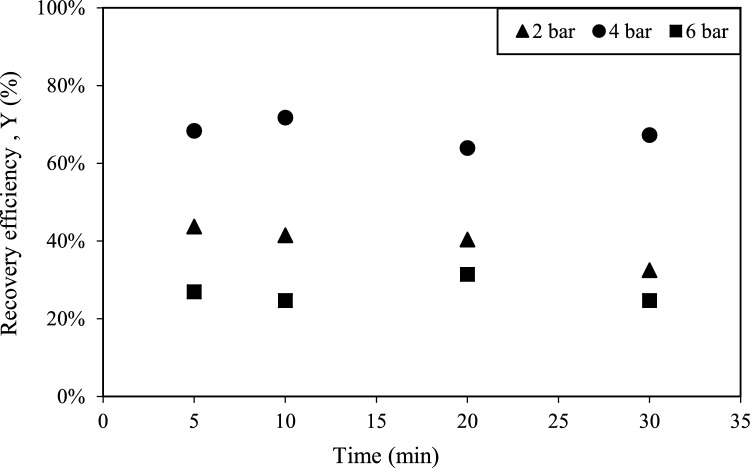
Effect of pressure on HC-assisted recovery efficiency with orifice-17

**Fig. 4:**
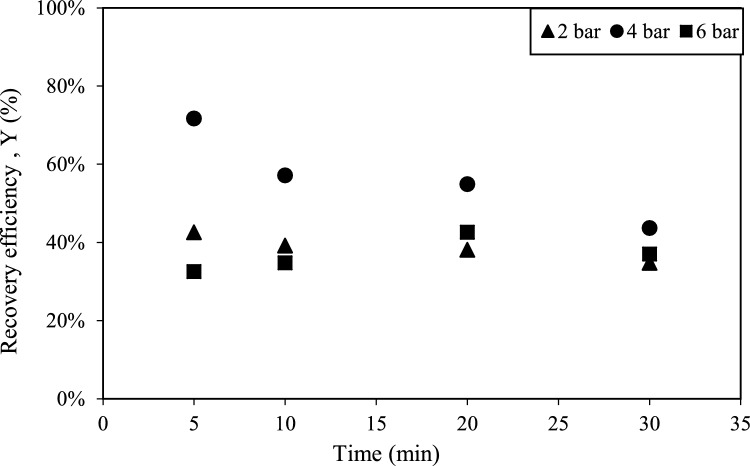
Effect of pressure on HC-assisted recovery efficiency with orifice-1

The effect of various pressure inputs (2, 4, and 6 bar), on efficiency of HC-assisted recovery was studied with orifice-1 and orifice-17 under constant solid load (0.5 g/L). Results are presented in Figs. 3 and 4 with respect to time. In order to examine the effect of interaction time on process efficiency, samples were collected every 5 min during the 30-min experiments.

Experiments conducted with two different orifice plates at various pressure inputs revealed no significant change in PHA recovery efficiency with increasing operation time. However, pressure played a crucial role in recovery performance, 4 bar yielding the highest efficiency for both orifice-1 and orifice-17.

As shown in Fig. 3, the highest efficiency (72%) was achieved after 10 min of operation using orifice-17. Increasing pressure from 2 to 4 bar led to an improvement in efficiency; however further increasing the pressure to 6 bar resulted in a significant decline, reducing recovery efficiency to 30%.

This trend can be explained by the role of upstream pressure in hydrodynamic cavitation (HC). At moderate pressures, higher shear forces enhance cavitation intensity, effectively disrupting bacterial cell walls and facilitating the release of intracellular PHA. However, at excessively high pressures, the merging of cavitation bubbles can lead to the formation of super cavitation, which collapses cavitational activity and reduces the efficiency of cell disruption [35, 36]. Thus, the observed decline in PHA recovery efficiency when increasing the pressure from 4 to 6 bar can be attributed to cavity coalescence, which disrupts optimal cavitation dynamics.

As illustrated in Fig. 4***,*** the maximum recovery efficiency (72%) was achieved after 5 min of operation with orifice-1 at pressure of 4 bar. While the recovery efficiency gradually declined over time at 2 bar and 4 bar, it initially increased at 6 bar, reaching its peak at 20 min before decreasing at 30 min.

The observed yield of 70% indicates that intracellular components may remain suspended, preventing their complete release from partially disrupted microbial cells. Additionally, the decline in recovery efficiency over extended processing times can be attributed to the progressive degradation of released PHBs, likely due to increased exposure to cavitational forces in the HC system [36].

Similar findings were reported by Xie et al., who observed that the levels of proteins and polysaccharides discharged from *B. subtilis* cells into suspension remained unchanged over time, supporting the hypothesis that extended cavitation may lead to further disintegration of previously released biopolymers rather than enhancing their recovery [36].

**Fig. 5:**
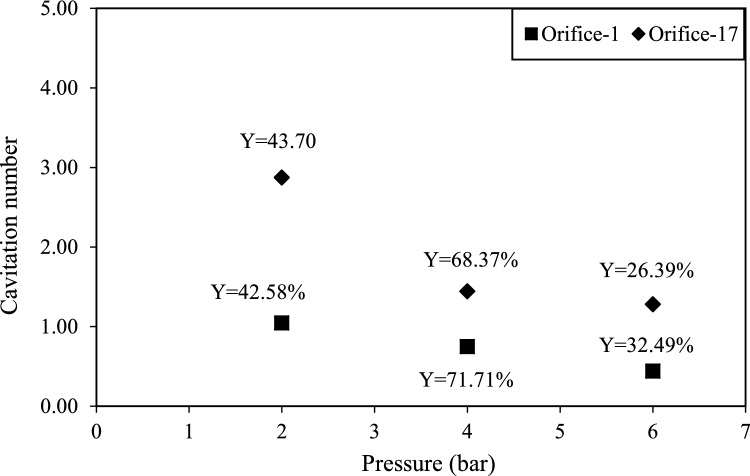
Effect of pressure and cavitation number on HC-assisted biopolymer recovery efficiency with different orifice plates

Selecting the optimal upstream pressure is critical in HC because it directly influences cavitation intensity, which is commonly evaluated using the CN. Previous studies suggest that a lower CN enhances cavitation activity and promoting greater cell disruption [37]. However, other studies have reported that the relationship between CN and the degree of cell disruption is not linear [19]. While significant cavitational effects are typically observed when CN is below 1, cavitation can still occur at higher CN values (2–4), potentially influenced by dissolved gases or impurities in the liquid medium [38]. In this study, orifice-17 exhibited a decrease in CN from 3.02 to 1.29 as pressure increased from 2 to 6 bar, whereas orifice-1 showed a CN of 1.03 at 2 bar, decreasing to 0.74 at 4 bar and 0.43 at 6 bar. Figure 5 demonstrates an apparent inverse relationship between CN and pressure for both types of orifices. Notably, optimal PHB recovery efficiencies for both orifice types were observed at a pressure of 4 bar, with values of 68.37% for orifice-17 and 71.71% for orifice-1. Although a lower CN is generally expected to enhance efficiency the observed decrease in efficiency at 6 bar pressure can be attributed to the overproduction of cavitation bubbles which may intensify the degradation of released PHBs. As cavitation continues throughout the cycle, the extracted PHBs may be subjected to further degradation, resulting an overall decline in recovery efficiency under high-pressure and low CN conditions.

### Statistical analysis

From the experimental results that were presented in Figs. 3 and 4, a quadratic equation was established to describe the HC-assisted biopolymer recovery in terms of actual factors for orifice-1 and orifice-17:

$${\text{Orifice-1}}:Y = - 0.279 \, + \, 0.4984 \, X2 \, - \, 0.00751 \, X3 \, - \, 0.06584 \, X2*X2 \, - \, 0.000044 \, X3*X3 \, + 0.001439 \, X2*X3$$

$${\text{Orifice-17}}:Y \, = \, - 0.211 \, + \, 0.4756 \, X2 \, - \, 0.00556 \, X3 \, - \, 0.06584 \, X2*X2 \, - \, 0.000044 \, X3*X3 \, + \, 0.001439 \, X2*X3$$

where *X*1, *X*2, *X*3, and *Y* represent the orifice type, pressure, operation time and resource recovery respectively. Table 1 shows the results from the analysis of variance (ANOVA), which evaluates the significance of the predictive model. The *p*-value of 0.000 shows that the overall model is highly significant.

**Table 1:** Analysis of variance (ANOVA) for response surface quadratic model

Source	DF	Adj SS	Adj MS	*F*-value	*p*-value
Model	8	0.422478	0.052810	12.33	0.000
Linear	3	0.026031	0.008677	2.03	0.153
*X*2	1	0.013374	0.013374	3.12	0.097
*X*3	1	0.011976	0.011976	2.80	0.115
*X*1	1	0.000682	0.000682	0.16	0.695
Square	2	0.370153	0.185076	43.23	0.000
*X*2*X2	1	0.369955	0.369955	86.40	0.000
*X*3*X3	1	0.000197	0.000197	0.05	0.833
2-way ınteraction	3	0.022688	0.007563	1.77	0.197
*X*2*X3	1	0.012211	0.012211	2.85	0.112
*X*2*X1	1	0.008362	0.008362	1.95	0.183
*X*3*X1	1	0.002115	0.002115	0.49	0.493
Error	15	0.064225	0.004282		
Total	23	0.486702			

Standard error of the regression (*S* = 0.0654343), coefficient of determination (*R*^2^ = 86.80%), adjusted *R*^2^ (*R*^2^ adj = 79.77%)

For the linear effects, the *p*-value of *X*2 (0.097) indicates that pressure is moderately significant but the *p*-values of *X*1, and *X*3 are greater than 0.1 indicated that the model terms used in the model were insignificant. For the quadratic effects, the term *X*2^2^ (Pressure^2^) has a highly significant *p*-value of 0.000, confirming that pressure has a nonlinear influence on efficiency.

**Fig. 6:**
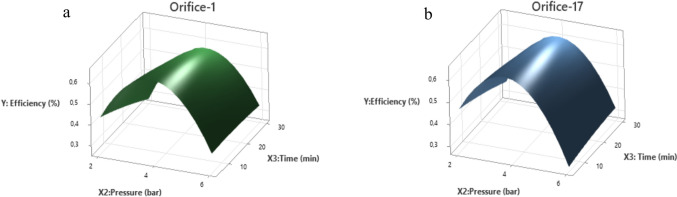
The surface response plots of the effects of pressure, time and orifice type. **a** Orifice-1, **b** orifice-17

To statistically validate the findings that were presented in Figs. 3 and 4 and analyze the interactions between independent variables, three-dimensional surface plots were constructed as shown in Fig. 6 for both orifice-1 and orifice-17. Both surface plots exhibit similar trend confirming insignificance of orifice type (*p* = 0.695 in ANOVA) and significant quadratic effect of pressure found in ANOVA. In both orifices, the optimal efficiency is achieved at moderate pressure (around 4 bar). Additionally, time has an observable effect on efficiency but not following a strong increasing or decreasing trend.

### Evaluation of biomass disruption mechanism under hydrodynamic cavitation

**Fig. 7:**
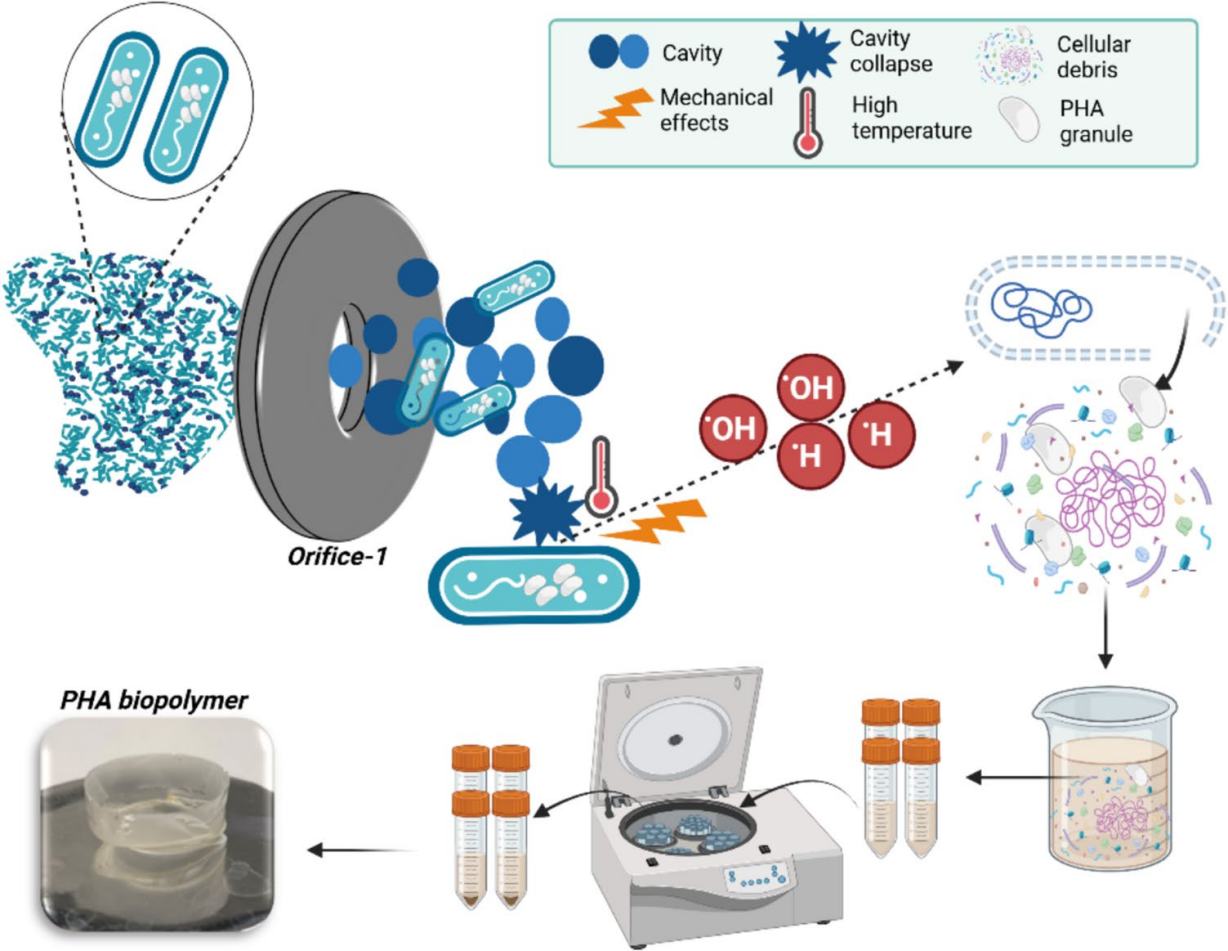
The mechanism of HC-assisted PHA recovery (Created in BioRender. Yılmaz (2025) https://BioRender.com/e924frd)

The HC reactor facilitates the passage of a suspension that contains PHA-containing MMC through its orifice. Acceleration of the liquid flow within the reactor induces a localized pressure reduction, which may lead to the formation of cavitation, provided that the pressure drop is lower than the saturation vapor pressure of the liquid. Mechanically, extreme conditions generated by the collapse of formed cavities produce effects such as shock waves, microjets of liquid, and high shear forces such as turbulence and vortices, as reported in the literature [17, 18, 21, 39]. Furthermore, the collapse of the cavities may lead to the generation of unpaired electrons, namely free radicals (^⋅^OH and ^⋅^H), within localized zones of notably elevated temperature. The various mechanical impacts induce bodily harm to the microorganism's external surface, leading to membrane disruption and the release of cellular constituents [40]. Since PHA is released together with other microbial cell components -such as cell debris, proteins, lipids and nucleic acids- during mechanical extraction methods, solvent use becomes necessary for its separation and purification from the resulting mixture. In this study, chloroform was selected due to its efficiency in dissolving and purifying PHA [41], enabling consistent polymer purity essential for downstream characterization and comparison. Specifically, chloroform was employed to dissolve and isolate PHA from a heterogeneous mixture of PHA and non-PHA cell mass (NPCM) in an aqueous medium. The mixture of chloroform, water, PHA, and NPCM was centrifuged to facilitate the precipitation of NPCM and the segregation of the chloroform phase containing the extracted biopolymer. Finally, the PHA biopolymer was recovered through chloroform evaporation. The mechanism of PHA recovery aided by HC is illustrated in Fig. 7.

**Fig. 8:**
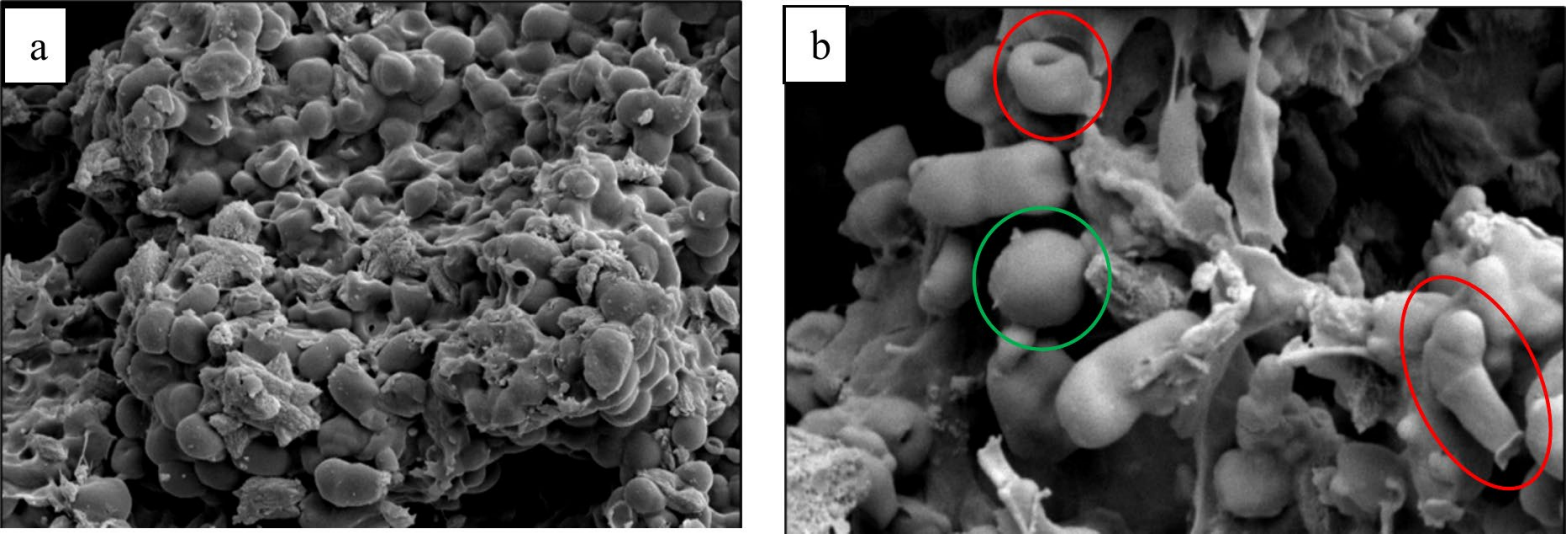
SEM images of biomass **a** before and **b** after cavitation at magnifications of × 5000 and × 10,000, respectively

To further support the proposed mechanism, bacterial cell morphology was analyzed before and after cavitation using scanning electron microscopy (SEM). Figure 8 presents SEM images of the biomass utilized in HC-assisted recovery, both before and after processing. The presence of MMC in the biomass posed a challenge in visualizing the microorganisms as uncontaminated culture images, similar to the observations reported by Khosravi-Darani et al. and Lee et al. [22, 42]. The SEM analysis demonstrated the undamaged state of the cells before HC treatment, which exhibited a spherical shape and clustered arrangement (Fig. 8a). Conversely, after HC recovery, the bacterial morphology underwent alterations, distinguished by distorted cell membranes and holes on the cell surface (Fig. 8b). Additionally, Fig. 8b highlights disrupted cells (marked in red circles) and intact cells (marked in green circles), providing visual confirmation of the HC-induced cell disruption process.

### Comparative evaluation of HC-assisted and solvent based PHB extraction

While solvent extraction achieved a higher PHB recovery yield (95.48%) and purity (83.69%) compared to HC-assisted extraction (71.71% yield, 71.40% purity), these results are expected given the well-established, aggressive cell-penetrating ability of chloroform.

In contrast, HC disrupts cells through localized high-pressure and temperature gradients, enabling mechanical disruption. Although this method may not rupture all cells completely—leading to a slightly lower molecular weight of PHB (0.15 × 10⁶ g/mol vs. 0.22 × 10⁶ g/mol)—it offers several significant advantages. These include simplified downstream processing, shorter operation times, and lower environmental impact.

### Chemical and thermal properties of recovered biopolymer

**Fig. 9:**
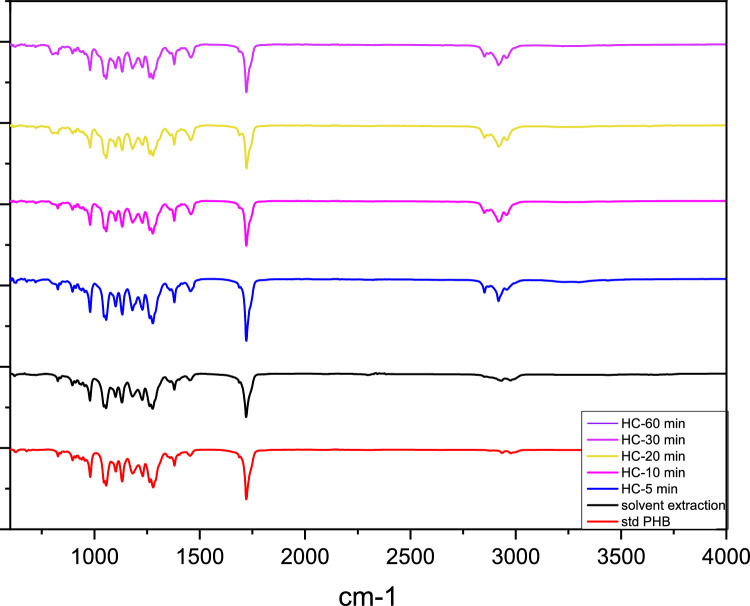
FTIR spectra of recovered polymers via HC under different operation times compared with standard PHB and chloroform-extracted biopolymer

The biopolymer sample recovered by the cavitation process with orifice-1 at 4 bar pressure after 5 min -the condition yielding the highest product efficiency—was selected for chemical and thermal characterization. Selected sample, derived from biomass via the HC method, displays promising attributes for commercial utilization. The measured purity of 71.4% indicates that the sample contains a significant proportion of PHB relative to impurities or other components. While higher purity is desirable for certain high-end applications, this level suggests that HC-assisted recovery process is effective in isolating PHB from biomass. Such purity may be adequate for less demanding uses, including biodegradable plastics for non-food packaging or agricultural applications. In addition to purity, molecular weight is a critical factor influencing mechanical strength and thermal stability. The measured molecular weight of 0.15 × 10⁶ g/mol falls within the typical range for PHB [43] confirming its suitability for applications requiring durable and thermally stable biopolymers. FTIR was employed to identify the presence of functional groups and confirm the PHB content of the extracted biopolymers. The FTIR spectra of commercial PHB (std PHB), and biopolymers that are recovered with HC process at different times were given in Fig. 9*.* To assess the effect of HC exposure time on the chemical structure, FTIR spectra were followed. It was observed that HC operation time had no effect on biopolymer’s chemical structure. PHB's characteristic peak was approximately 1720 cm^−1^ (C=O stretch), and the ester group's other strong peaks were between 1000 and 1400 cm^−1^ (C–O stretch). [44]. All extracted biopolymers were compatible with commercial PHB.

**Fig. 10:**
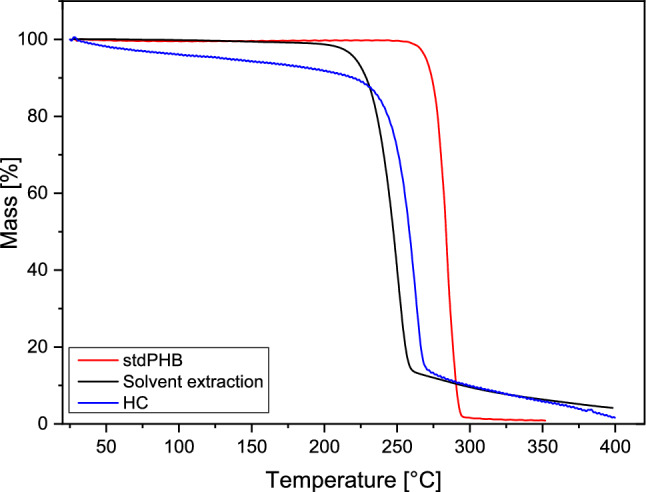
TGA of recovered biopolymers by HC comparison with std PHB

The thermal degradation profiles for commercial PHB and extracted biopolymers were shown in Fig. 10***.*** Results showed that the thermal stability of the recovered biopolymer differed slightly from that of the commercial standard. While the initial degradation starts at 269.75 °C for commercial PHB, it starts at 243.21 °C for HC recovery and 225.83 °C for chloroform extraction. It was seen that HC-assisted extracted biopolymers’ maximum thermal degradation (262.12 °C) was closer to the chloroform extracted biopolymer (261.44 °C) and lower than the commercial PHB (296.62 °C).

PHBs obtained through HC process exhibited a lower resistance to deterioration at lower temperatures compared to commercial PHB. In the HC-recovered PHB, a 10% mass loss was observed at temperatures up to 243.21 °C. This may be attributed to the evaporation of residual solvents physically adsorbed in the PHB recovered via the HC process [45]. It was seen that HC-assisted extracted biopolymers’ maximum thermal degradation (262.12 °C) were slightly lower than the commercial PHB (296.62 °C). The data presented in the figure indicates that following the initial thermal decomposition, approximately 20% of the original mass recovered from microorganisms by HC process remains between 250 and 400 °C. The presence of residual mass in the PHB matrix is likely due to impurities, particularly lignin residues that were not metabolized and remained stable within the thermal degradation ranges [46]. Residual impurities may be removed or reduced through upstream methods such as NaOCl or NaOH pretreatment of biomass, or downstream approaches including biopolymer precipitation using ethanol or methanol, re-dissolution in solvents like 1-butanol or 2-propanol, ozonation and activated charcoal filtration [41].

## Conclusion

This study introduces a novel technique for separating and recovering PHA from MMC biomass, employing HC for the first time to disrupt microbial cells and release intracellular PHA.

The recovered biopolymers were found to be chemically and thermally compatible with commercial PHB. With extraction efficiency, purity, and molecular weight comparable to conventional solvent extraction, the HC-assisted method offers notable advantages, including simplified downstream processing, reduced operation time, and a lower environmental impact. While chloroform was used in minimal amounts for this study, future research will focus on integrating more sustainable solvents to enhance the method’s environmental performance.

HC shows strong potential for industrial PHA recovery due to its efficient cell disruption, low energy use, and simple, cost-effective components, which lead to lower capital investment and reduced chemical and thermal requirements. While further research is needed to address scale-up challenges, alternative cavitation devices—such as vortex or rotor–stator reactors—may improve feasibility. Although no economic analysis was performed here, future research should focus on cost evaluation and process optimization to confirm industrial viability.

## Data Availability

No datasets were generated or analysed during the current study.
